# Supplementary material for: Accurate prediction of kinase-substrate networks using knowledge graphs
Source: PLoS Comput Biol. 2020 Dec 3;16(12):e1007578. doi: 10.1371/journal.pcbi.1007578 (PMC7738173; doi:10.1371/journal.pcbi.1007578)
Supplement: S1 Table — (PDF) [file pcbi.1007578.s001.pdf]

Supplementary Table 1: PKA Kinase Assay Results

Note: The * symbol in the **Predicted** column of all tables indicates whether or not the substrates were present at all in the LinkPhinder data. The supporting mass spec data for these results is provided in the Supplementary Tables 5, 6 and 7.

**Showing the identified substrates of PKA**			
**Protein IDs**	**Protein names**	**Gene names**	**Predicted**
P51665	26S proteasome non-ATPase regulatory subunit 7	PSMD7	No
P21980	Protein-glutamine gamma-glutamyltransferase 2	TGM2	Yes*
Q5T749	Keratinocyte proline-rich protein	KPRP	No
P60468	Protein transport protein Sec61 subunit beta	SEC61B	No
Q6UWP8	Suprabasin	SBSN	No
P04259	Keratin, type II cytoskeletal 6B	KRT6B	No
K7EJC1	26S proteasome non-ATPase regulatory subunit 8	PSMD8	No
P10599	Thioredoxin	TXN	No*
E7EQL5	Cytoplasmic dynein 1 intermediate chain 2	DYNC1I2	No
P62195	26S protease regulatory subunit 8	PSMC5	Yes*
E9PLL6	60S ribosomal protein L27a	RPL27A	No
Q5JNZ5	Putative 40S ribosomal protein S26-like 1	RPS26P11	No
Q08554	Desmocollin-1	DSC1	No
B0YIW6	Coatomer subunit delta	ARCN1	No
I3L0H8	ATP-dependent RNA helicase DDX19A	DDX19A	No
O76094	Signal recognition particle subunit SRP72	SRP72	No
F5GY37	Prohibitin-2	PHB2	No
M0QXM4	Amino acid transporter	SLC1A5	No
D6RHZ5	Protein transport protein Sec31A	SEC31A	No
Q02413	Desmoglein-1	DSG1	No
P51572	B-cell receptor-associated protein 31	BCAP31	No
P31944	Caspase-14	CASP14	No
O95373	Importin-7	IPO7	No
Q9UQ80	Proliferation-associated protein 2G4	PA2G4	Yes*
H3BV80	RNA-binding protein with serine-rich domain 1	RNPS1	No
M0R3D6	60S ribosomal protein L18a	RPL18A	No
X6RFL8	Ras-related protein Rab-14	RAB14	No

Continued on next page

Showing the identified substrates of PKA (cont.)			
**Protein IDs**	**Protein names**	**Gene names**	**Predicted**
P02647	Apolipoprotein A-I	APOA1	No
P48643	T-complex protein 1 subunit epsilon	CCT5	No
Q13283	Ras GTPase-activating protein-binding protein 1	G3BP1	No*
P17612	cAMP-dependent protein kinase catalytic subunit alpha	PRKACA	Yes*
A0A087X0K8	Probable G-protein coupled receptor 179	GPR179	No
P13667	Protein disulfide-isomerase A4	PDIA4	No
Q9Y2Z0	Suppressor of G2 allele of SKP1 homolog	SUGT1	No*
