# Supplementary material for: Accurate prediction of kinase-substrate networks using knowledge graphs
Source: PLoS Comput Biol. 2020 Dec 3;16(12):e1007578. doi: 10.1371/journal.pcbi.1007578 (PMC7738173; doi:10.1371/journal.pcbi.1007578)
Supplement: S2 Table — (PDF) [file pcbi.1007578.s002.pdf]

Supplementary Table 2: MST2 Kinase Assay Results

Note: The * symbol in the **Predicted** column of all tables indicates whether or not the substrates were present at all in the LinkPhinder data. The supporting mass spec data for these results is provided in the Supplementary Tables 5, 6 and 7.

**Showing the identified substrates of MST2**			
**Protein IDs**	**Protein names**	**Gene names**	**Predicted**
H7C0Q6	E3 ubiquitin-protein ligase TRIM4	TRIM4	No
P35606	Coatomer subunit beta	COPB2	No
H3BPE7	RNA-binding protein FUS	FUS	No
P26196	Probable ATP-dependent RNA helicase DDX6	DDX6	No
P56385	ATP synthase subunit e, mitochondrial	ATP5I	No
Q5SRQ6	Casein kinase II subunit beta	CSNK2B	No
O95801	Tetratricopeptide repeat protein 4	TTC4	No
Q49AN9	Small nuclear ribonucleoprotein G	SNRPG	No
D6RAW0	Ubiquitin-conjugating enzyme E2 D2	UBE2D3	No
O15145	Actin-related protein 2/3 complex subunit 3	ARPC3	No
Q13188	Serine/threonine-protein kinase 3	STK3	Yes*
H7C3H9	Methyltransferase-like protein 6	METTL6	No
O15212	Prefoldin subunit 6	PFDN6	No
E7EQG2	Eukaryotic initiation factor 4A-II	EIF4A2	No
E5RHG8	Transcription elongation factor B polypeptide 1	TCEB1	No
O15355	Protein phosphatase 1G	PPM1G	No*
K7EJL1	AP-1 complex subunit mu-1	AP1M1	No
H7BXH2	Serine/threonine-protein phosphatase 6 regulatory subunit 3	PPP6R3	No
H3BV80	RNA-binding protein with serine-rich domain 1	RNPS1	No
P36543	V-type proton ATPase subunit E 1	ATP6V1E1	No
Q9Y2Q3	Glutathione S-transferase kappa 1	GSTK1	No
Q59GN2	Putative 60S ribosomal protein L39-like 5	RPL39P5	No
Q96M27	Protein PRRC1	PRRC1	No
P17858	ATP-dependent 6-phosphofructokinase, liver type	PFKL	No
B1AJY5	26S proteasome non-ATPase regulatory subunit 10	PSMD10	No

Continued on next page

Showing the identified substrates of MST2 (cont.)			
**Protein IDs**	**Protein names**	**Gene names**	**Predicted**
Q9Y2H2	Phosphatidylinositide phosphatase SAC2	INPP5F	No
B5MC59	Replication protein A 14 kDa subunit	RPA3	No
E7EMD0	NADPH–cytochrome P450 reductase	POR	No
E7ET15	U2 snRNP-associated SURP motif-containing protein	U2SURP	No
D6RBW1	Eukaryotic translation initiation factor 4E	EIF4E	No
D6RBV2	Vesicular integral-membrane protein VIP36	LMAN2	No
P32322	Pyrroline-5-carboxylate reductase 1, mitochondrial	PYCR1	No*
Q6NUK1	Calcium-binding mitochondrial carrier protein SCaMC-1	SLC25A24	No
H0YJU3	DNA mismatch repair protein Mlh3	MLH3	No
P35250	Replication factor C subunit 2	RFC2	No
K7ER96	Thioredoxin-like protein 1	TXNL1	No
Q96I24	Far upstream element-binding protein 3	FUBP3	No
V9GZ61	28S ribosomal protein S29, mitochondrial	DAP3	No
F8W8Z9	Mitochondrial import receptor subunit TOM5 homolog	TOMM5	No
Q9H0C8	Integrin-linked kinase-associated serine/threonine phosphatase 2C	ILKAP	No
A0A087-WY71	AP-2 complex subunit mu	AP2M1	No
Q9H8S9	MOB kinase activator 1A	MOB1A	Yes*
A0A087-WZN1	Isocitrate dehydrogenase [NAD] subunit, mitochondrial	IDH3B	No
C9J3L8	Translocon-associated protein subunit alpha	SSR1	No
Q15417	Calponin-3	CNN3	No*
Q9BSJ8	Extended synaptotagmin-1	ESYT1	No
J3KQ96	Treacle protein	TCOF1	No
Q9Y2Z4	Tyrosine–tRNA ligase, mitochondrial	YARS2	No
Q96I99	Succinyl-CoA ligase [GDP-forming] subunit beta, mitochondrial	SUCLG2	No
B8ZZQ4	Pentatricopeptide repeat domain-containing protein 3, mitochondrial	PTCD3	No
Q53EL6	Programmed cell death protein 4	PDCD4	No*
B1AK87	F-actin-capping protein subunit beta	CAPZB	No

Continued on next page

Showing the identified substrates of MST2 (cont.)			
**Protein IDs**	**Protein names**	**Gene names**	**Predicted**
Q9BXW7	Cat eye syndrome critical region protein 5	CECR5	No
P51148	Ras-related protein Rab-5C	RAB5C	No
P27144	Adenylate kinase 4, mitochondrial	AK4	No
P48634	Protein PRRC2A	PRRC2A	No*
E9PRB9	Signal peptidase complex subunit 2	SPCS2	No
O43491	Band 4.1-like protein 2	EPB41L2	No
E9PH64	NADH dehydrogenase [ubiquinone] 1 beta subcomplex subunit 9	NDUFB9	No
E7EX90	Dynactin subunit 1	DCTN1	No
A0A0A0-MTN3	Glutathione S-transferase Mu 3	GSTM3	No
Q15746	Myosin light chain kinase, smooth muscle	MYLK	No*
P51970	NADH dehydrogenase [ubiquinone] 1 alpha subcomplex subunit 8	NDUFA8	No
J3KSC4	Ras-related C3 botulinum toxin substrate 3	RAC3	No
B4DR61	Protein transport protein Sec61 subunit alpha isoform 1	SEC61A1	No
P09972	Fructose-bisphosphate aldolase C	ALDOC	No
P42765	3-ketoacyl-CoA thiolase, mitochondrial	ACAA2	No
Q96AT9	Ribulose-phosphate 3-epimerase	RPE	No
A0A0B4J296	Transcription elongation factor B polypeptide 2	TCEB2	No
C9JG32	Elongation factor Ts	TSFM	No
Q9NT62	Ubiquitin-like-conjugating enzyme ATG3	ATG3	No
A0AVT1	Ubiquitin-like modifier-activating enzyme 6	UBA6	No
H0YAT2	28S ribosomal protein S28, mitochondrial	MRPS28	No
Q9Y2S7	Polymerase delta-interacting protein 2	POLDIP2	No
P28288	ATP-binding cassette sub-family D member 3	ABCD3	No
A0A1B0-GWE0	Dedicator of cytokinesis protein 7	DOCK7	No
O95292	Vesicle-associated membrane protein-associated protein B/C	VAPB	No
O00767	Acyl-CoA desaturase	SCD	No
U3KQC1	WD repeat-containing protein 18	WDR18	No
Q96GA3	Protein LTV1 homolog	LTV1	No

Continued on next page

Showing the identified substrates of MST2 (cont.)			
**Protein IDs**	**Protein names**	**Gene names**	**Predicted**
Q9Y570	Protein phosphatase methylesterase 1	PPME1	No*
Q5VYD6	HCLS1-associated protein X-1	HAX1	No
F5GX39	Transmembrane emp24 domain-containing protein 2	TMED2	No
Q9ULX3	RNA-binding protein NOB1	NOB1	No*
G3V2G6	Retinol dehydrogenase 11	RDH11	No
Q96PU8	Protein quaking	QKI	No
Q92620	Pre-mRNA-splicing factor ATP-dependent RNA helicase PRP16	DHX38	No*
A0A0A0-MT33	Protein SCAF8	SCAF8	No
H0YEF3	Ribonuclease H2 subunit C	RNASEH2C	No
Q9Y399	28S ribosomal protein S2, mitochondrial	MRPS2	No
A0A0J9-YXM1	Serum paraoxonase/arylesterase 2	PON2	No
H0YEL5	Peptidyl-prolyl cis-trans isomerase	PPIH	No
O00505	Importin subunit alpha-4	KPNA3	No
C9IZ01	Elongation factor G, mitochondrial	GFM1	No
H7BYZ9	Mitochondrial amidoxime-reducing component 1	Mar-01	No
P61604	10 kDa heat shock protein, mitochondrial	HSPE1	No
Q8TD19	Serine/threonine-protein kinase Nek9	NEK9	No*
O00743	Serine/threonine-protein phosphatase 6 catalytic subunit	PPP6C	No
Q9NUQ9	Protein FAM49B	FAM49B	No
Q7Z7E8	Ubiquitin-conjugating enzyme E2 Q1	UBE2Q1	No
O43592	Exportin-T	XPOT	No
Q9Y248	DNA replication complex GINS protein PSF2	GINS2	No*
Q96ER9	Coiled-coil domain-containing protein 51	CCDC51	No
G5E9W3	Cleavage and polyadenylation specificity factor subunit 3	CPSF3	No
E9PL37	Calpain-1 catalytic subunit	CAPN1	No
Q12972	Nuclear inhibitor of protein phosphatase 1	PPP1R8	No*
A0A140T9T4	Putative pre-mRNA-splicing factor ATP-dependent RNA helicase DHX16	DHX16	No

Continued on next page

Showing the identified substrates of MST2 (cont.)			
**Protein IDs**	**Protein names**	**Gene names**	**Predicted**
P35573	Glycogen debranching enzyme	AGL	No
O60563	Cyclin-T1	CCNT1	No*
E7EPW2	28S ribosomal protein S25, mitochondrial	MRPS25	No
Q9H857	5-nucleotidase domain-containing protein 2	NT5DC2	No
Q9UKG1	DCC-interacting protein 13-alpha	APPL1	No*
Q9H993	Protein-glutamate O-methyltransferase	ARMT1	No
H7C2P7	39S ribosomal protein L23, mitochondrial	MRPL23	No
P36404	ADP-ribosylation factor-like protein 2	ARL2	No
H0YMD0	Annexin	ANXA2	No
Q5QPN5	Acyl-protein thioesterase 2	LYPLA2	No
O43747	AP-1 complex subunit gamma-1	AP1G1	No
Q969G6	Riboflavin kinase	RFK	No
O00178	GTP-binding protein 1	GTPBP1	No
Q96HE7	ERO1-like protein alpha	ERO1L	No
Q15121	Astrocytic phosphoprotein PEA-15	PEA15	No*
G3XAL9	Solute carrier family 12 member 2	SLC12A2	No
I3L2N2	Segment polarity protein dishevelled homolog DVL-2	DVL2	No
Q15437	Protein transport protein Sec23B	SEC23B	No
Q15404	Ras suppressor protein 1	RSU1	No
B5MC98	Prolactin regulatory element-binding protein	PREB	No
Q9BS26	Endoplasmic reticulum resident protein 44	ERP44	No
E9PKV2	39S ribosomal protein L17, mitochondrial	MRPL17	No
Q9P0J0	NADH dehydrogenase [ubiquinone] 1 alpha subcomplex subunit 13	NDUFA13	No
Q9UBF2	Coatomer subunit gamma-2	COPG2	No
G3V2E7	Kinesin light chain 1	KLC1	No
Q12797	Aspartyl/asparaginyl beta-hydroxylase	ASPH	No
Q5T9A4	ATPase family AAA domain-containing protein 3B	ATAD3B	No
A0A0C4-DGA6	Helicase-like transcription factor	HLTF	No
Q15120	[Pyruvate dehydrogenase (acetyl-transferring)] kinase isozyme 3, mitochondrial	PDK3	No

Continued on next page

Showing the identified substrates of MST2 (cont.)			
**Protein IDs**	**Protein names**	**Gene names**	**Predicted**
G5E9F5	Protein Mpv17	MPV17	No
F5H4B6	Aldehyde dehydrogenase family 16 member A1	ALDH16A1	No
F5H8D7	DNA repair protein XRCC1	XRCC1	No
O95340	Bifunctional 3-phosphoadenosine 5-phosphosulfate synthase 2	PAPSS2	No
C9IZ83	DnaJ homolog subfamily C member 2	DNAJC2	No
O43252	Bifunctional 3-phosphoadenosine 5-phosphosulfate synthase 1	PAPSS1	No
P61088	Ubiquitin-conjugating enzyme E2 N	UBE2N	No
F6RFD5	Destrin	DSTN	No
Q969Z0	Protein TBRG4	TBRG4	No
J3KQ32	Obg-like ATPase 1	OLA1	No
P51812	Ribosomal protein S6 kinase alpha-3	RPS6KA3	No*
J3KTL8	Structural maintenance of chromosomes flexible hinge domain-containing protein 1	SMCHD1	No
H0Y5Q7	Isocitrate dehydrogenase [NAD] subunit, mitochondrial	IDH3G	No
O14980	Exportin-1	XPO1	No*
Q99536	Synaptic vesicle membrane protein VAT-1 homolog	VAT1	No
P50402	Emerin	EMD	No*
D6RAA6	Transmembrane protein 33	TMEM33	No
Q12874	Splicing factor 3A subunit 3	SF3A3	No
Q8N8S7	Protein enabled homolog	ENAH	No
A0A024R4E5	Vigilin	HDLBP	No
Q9UHD8	Septin-9	Sep-09	No
P49790	Nuclear pore complex protein Nup153	NUP153	Yes*
Q13492	Phosphatidylinositol-binding clathrin assembly protein	PICALM	No
Q9NTZ6	RNA-binding protein 12	RBM12	No
M0QX71	Glutamate-rich WD repeat-containing protein 1	GRWD1	No
Q13769	THO complex subunit 5 homolog	THOC5	No*
P30040	Endoplasmic reticulum resident protein 29	ERP29	No
Q9NVJ2	ADP-ribosylation factor-like protein 8B	ARL8B	No

Continued on next page

Showing the identified substrates of MST2 (cont.)			
**Protein IDs**	**Protein names**	**Gene names**	**Predicted**
O43813	LanC-like protein 1	LANCL1	No
Q15459	Splicing factor 3A subunit 1	SF3A1	No
Q8N5M9	Protein jagunal homolog 1	JAGN1	No
P19784	Casein kinase II subunit alpha	CSNK2A2	No
P49711	Transcriptional repressor CTCF	CTCF	No
A0A0A0-MRN7	Kynurenine–oxoglutarate transaminase 3	CCBL2	No
O60664	Perilipin-3	PLIN3	No
Q9C0C9	E2/E3 hybrid ubiquitin-protein ligase UBE2O	UBE2O	No*
Q5T6H7	Xaa-Pro aminopeptidase 1	XPNPEP1	No
E9PB90	Hexokinase	HK2	No
E7ETK0	40S ribosomal protein S24	RPS24	No
P09496	Clathrin light chain A	CLTA	No
A0A0C4-DGX4	Cullin-1	CUL1	No
O14929	Histone acetyltransferase type B catalytic subunit	HAT1	No
Q9HAV7	GrpE protein homolog 1, mitochondrial	GRPEL1	No
B1AH49	Sulfurtransferase	MPST	No
P36871	Phosphoglucomutase-1	PGM1	No*
O95295	SNARE-associated protein Snapin	SNAPIN	Yes*
P49257	Protein ERGIC-53	LMAN1	No
Q15126	Phosphomevalonate kinase	PMVK	No
O43390	Heterogeneous nuclear ribonucleoprotein R	HNRNPR	No
A0A1W2-PNX8	Protein unc-45 homolog A	UNC45A	No
O43719	HIV Tat-specific factor 1	HTATSF1	No
P61011	Signal recognition particle 54 kDa protein	SRP54	No
Q9NR45	Sialic acid synthase	NANS	No
Q15136	cAMP-dependent protein kinase catalytic subunit alpha	KIN27	No
Q15029	116 kDa U5 small nuclear ribonucleoprotein component	EFTUD2	No
A0A0A0-MRA5	Heterogeneous nuclear ribonucleoprotein U-like protein 1	HNRNPUL1	No

Continued on next page

Showing the identified substrates of MST2 (cont.)			
**Protein IDs**	**Protein names**	**Gene names**	**Predicted**
Q99575	Ribonucleases P/MRP protein subunit POP1	POP1	No
O60493	Sorting nexin-3	SNX3	No
K7EP07	Tubulin-folding cofactor B	TBCB	No
P25788	Proteasome subunit alpha type-3	PSMA3	No
Q9Y295	Developmentally-regulated GTP-binding protein 1	DRG1	No*
P62820	Ras-related protein Rab-1A	RAB1A	No*
E7EX17	Eukaryotic translation initiation factor 4B	EIF4B	No
Q5T8P6	RNA-binding protein 26	RBM26	No*
P08240	Signal recognition particle receptor subunit alpha	SRPR	No
Q96F86	Enhancer of mRNA-decapping protein 3	EDC3	No*
P61970	Nuclear transport factor 2	NUTF2	No
Q9UDW1	Cytochrome b-c1 complex subunit 9	UQCR10	No
A0A0A0-MSJ0	ATP-dependent RNA helicase DDX42	DDX42	No
P00505	Aspartate aminotransferase, mitochondrial	GOT2	No
Q15691	Microtubule-associated protein RP/EB family member 1	MAPRE1	No*
H0YDN9	ADP-ribosylation factor GTPase-activating protein 2	ARFGAP2	No
O00232	26S proteasome non-ATPase regulatory subunit 12	PSMD12	No
Q8TEX9	Importin-4	IPO4	No
A2AB27	Guanine nucleotide-binding protein-like 1	GNL1	No
