# Supplementary material for: Accurate prediction of kinase-substrate networks using knowledge graphs
Source: PLoS Comput Biol. 2020 Dec 3;16(12):e1007578. doi: 10.1371/journal.pcbi.1007578 (PMC7738173; doi:10.1371/journal.pcbi.1007578)
Supplement: S3 Table — (PDF) [file pcbi.1007578.s003.pdf]

Supplementary Table 1: LATS1 Kinase Assay Results

Note: The * symbol in the **Predicted** column of all tables indicates whether or not the substrates were present at all in the LinkPhinder data. The supporting mass spec data for these results is provided in the Supplementary Tables 5, 6 and 7.

**Showing the identified substrates of LATS1**			
**Protein IDs**	**Protein names**	**Gene names**	**Predicted**
H7C1J4	UHRF1-binding protein 1	UHRF1BP1	No
M0QZJ5	Calcium-binding mitochondrial carrier protein SCaMC-3	SLC25A23	No
R4GN98	Protein S100	S100A6	No
P08559	Pyruvate dehydrogenase E1 component subunit alpha, somatic form, mitochondrial	PDHA1	No*
P50995	Annexin A11	ANXA11	No
A6NLN1	Polypyrimidine tract-binding protein 1	PTBP1	No
P30048	Thioredoxin-dependent peroxide reductase, mitochondrial	PRDX3	No
P00387	NADH-cytochrome b5 reductase 3	CYB5R3	No
A0A1W2-PRU0	Alpha-endosulfine	ENSA	No
K7ENI6	Transmembrane protein 256	TMEM256	No
P00167	Cytochrome b5	CYB5A	No
H0YN26	Acidic leucine-rich nuclear phosphoprotein 32 family member A	ANP32A	No
O95835	Serine/threonine-protein kinase LATS1	LATS1	Yes*
P62899	60S ribosomal protein L31	RPL31	No
Q16352	Alpha-internexin	INA	No
A0A075B7B8	Immunoglobulin heavy variable 3/OR16-12	IGHV3OR16-12	No
Q5H8X8	Urotensin-2	UTS2	No
J3KR24	Isoleucine–tRNA ligase, cytoplasmic	IARS	No
Q5QNZ2	ATP synthase F(0) complex subunit B1, mitochondrial	ATP5F1	No
P46778	60S ribosomal protein L21	RPL21	No*
J3QR09	Ribosomal protein L19	RPL19	No
E9PR30	40S ribosomal protein S30	FAU	No

Continued on next page

Showing the identified substrates of LATS1 (cont.)			
**Protein IDs**	**Protein names**	**Gene names**	**Predicted**
Q07021	Complement component 1 Q subcomponent-binding protein, mitochondrial	C1QBP	No
P35637	RNA-binding protein FUS	FUS	No*
P53396	ATP-citrate synthase	ACLY	No
A0A087-WXM6	60S ribosomal protein L17	RPL17	No
J3QL05	Serine/arginine-rich splicing factor 2	SRSF2	No
P60891	Ribose-phosphate pyrophosphokinase 1	PRPS1	No
Q8WW12	PEST proteolytic signal-containing nuclear protein	PCNP	No
Q9Y696	Chloride intracellular channel protein 4	CLIC4	No
E9PQ63	Carbonyl reductase [NADPH] 1	CBR1	No
Q5T446	Uroporphyrinogen decarboxylase	UROD	No
E5RID6	2,4-dienoyl-CoA reductase, mitochondrial	DECR1	No
P31415	Calsequestrin-1	CASQ1	No
M0QX65	SUMO-activating enzyme subunit 1	SAE1	No
Q9BUF5	Tubulin beta-6 chain	TUBB6	No
P09211	Glutathione S-transferase P	GSTP1	No*
Q08211	ATP-dependent RNA helicase A	DHX9	No
A6NJA2	Ubiquitin carboxyl-terminal hydrolase	USP14	No
H0Y8E6	DNA replication licensing factor MCM2	MCM2	No
C9J2C4	DnaJ homolog subfamily B member 6	DNAJB6	No
Q15427	Splicing factor 3B subunit 4	SF3B4	No
A0A0U1-RQF0	Fatty acid synthase	FASN	No
O75746	Calcium-binding mitochondrial carrier protein Aralar1	SLC25A12	No
P31153	S-adenosylmethionine synthase isoform type-2	MAT2A	No
C9JBL1	Signal peptidase complex subunit 1	SPCS1	No
P36542	ATP synthase subunit gamma, mitochondrial	ATP5C1	No*
E9PMH2	AH receptor-interacting protein	AIP	No
O43390	Heterogeneous nuclear ribonucleoprotein R	HNRNPR	No

Continued on next page

Showing the identified substrates of LATS1 (cont.)			
**Protein IDs**	**Protein names**	**Gene names**	**Predicted**
P67775	Serine/threonine-protein phosphatase 2A catalytic subunit alpha isoform	PPP2CA	No*
J3KQ48	Peptidyl-tRNA hydrolase 2, mitochondrial	PTRH2	No
O43583	Density-regulated protein	DENR	No
J3QQM1	26S protease regulatory subunit 8	PSMC5	No
Q9Y265	RuvB-like 1	RUVBL1	No*
P61586	Transforming protein RhoA	RHOA	Yes*
E9PPQ5	Cysteine and histidine-rich domain-containing protein 1	CHORDC1	No
A6NNI4	Tetraspanin	CD9	No
Q13561	Dynactin subunit 2	DCTN2	No
A6NHL2	Tubulin alpha chain-like 3	TUBAL3	No
P08243	Asparagine synthetase [glutamine-hydrolyzing]	ASNS	No
O95202	LETM1 and EF-hand domain-containing protein 1, mitochondrial	LETM1	No
E7EST3	Tetratricopeptide repeat protein 9C	TTC9C	No
P48735	Isocitrate dehydrogenase [NADP], mitochondrial	IDH2	No
P42166	Lamina-associated polypeptide 2, isoform alpha	TMPO	No*
P10606	Cytochrome c oxidase subunit 5B, mitochondrial	COX5B	No
P02747	Complement C1q subcomponent subunit C	C1QC	No
Q9H773	dCTP pyrophosphatase 1	DCTPP1	No
P02585	Troponin C, skeletal muscle	TNNC2	No
P07360	Complement component C8 gamma chain	C8G	No
F8WF15	Ubiquitin-conjugating enzyme E2 E2	UBE2E2	No
X6RLJ0	Complement C1q subcomponent subunit A	C1QA	No
E7EPA1	Phosphoribosyl pyrophosphate synthase-associated protein 2	PRPSAP2	No
Q14166	Tubulin–tyrosine ligase-like protein 12	TTLL12	No
Q9H3P7	Golgi resident protein GCP60	ACBD3	No
Q92688	Acidic leucine-rich nuclear phosphoprotein 32 family member B	ANP32B	No*

Continued on next page

Showing the identified substrates of LATS1 (cont.)			
**Protein IDs**	**Protein names**	**Gene names**	**Predicted**
I3L471	Phosphatidylinositol transfer protein alpha isoform	PITPNA	No
E9PQP1	NADH dehydrogenase [ubiquinone] flavoprotein 1, mitochondrial	NDUFV1	No
C9JLU1	DNA-directed RNA polymerases I, II, and III subunit RPABC3	POLR2H	No
H7C402	Proteasome subunit alpha type	PSMA2	No
K7EQ02	DAZ-associated protein 1	DAZAP1	No
P23634	Plasma membrane calcium-transporting ATPase 4	ATP2B4	No
H0YBW4	Phospholipase A-2-activating protein	PLAA	No
H0YK48	Tropomyosin alpha-1 chain	TPM1	No
P53007	Tricarboxylate transport protein, mitochondrial	SLC25A1	No
J3KRT0	Core-binding factor subunit beta	CBFB	No
F8WBH7	Proteasome assembly chaperone 1	PSMG1	No
A0A087-WUD3	Oligosaccharyltransferase complex subunit OSTC	OSTC	No
A0A087X054	Hypoxia up-regulated protein 1	HYOU1	No
Q71U36	Tubulin alpha-1A chain	TUBA1A	No
E9PBC5	Plasma kallikrein	KLKB1	No
Q92905	COP9 signalosome complex subunit 5	COPS5	No
Q8NC51	Plasminogen activator inhibitor 1 RNA-binding protein	SERBP1	No
P49915	GMP synthase [glutamine-hydrolyzing]	GMPS	No*
P31949	Protein S100-A11	S100A11	No
O75369	Filamin-B	FLNB	No
P61313	60S ribosomal protein L15	RPL15	No
Q14677	Clathrin interactor 1	CLINT1	No
P34897	Serine hydroxymethyltransferase, mitochondrial	SHMT2	No
Q13151	Heterogeneous nuclear ribonucleoprotein A0	HNRNPA0	No*
P39023	60S ribosomal protein L3	RPL3	No
P31930	Cytochrome b-c1 complex subunit 1, mitochondrial	UQCRC1	No

Continued on next page

Showing the identified substrates of LATS1 (cont.)			
**Protein IDs**	**Protein names**	**Gene names**	**Predicted**
P62913	60S ribosomal protein L11	RPL11	No*
B7ZAR1	T-complex protein 1 subunit epsilon	CCT5	No
P54577	Tyrosine–tRNA ligase, cytoplasmic	YARS	No
H0Y4R1	Inosine-5-monophosphate dehydrogenase 2	IMPDH2	No
Q15181	Inorganic pyrophosphatase	PPA1	No
A0A087-WZ51	Tumor protein D54	TPD52L2	No
Q9BWD1	Acetyl-CoA acetyltransferase, cytosolic	ACAT2	No
P24752	Acetyl-CoA acetyltransferase, mitochondrial	ACAT1	No*
Q9H444	Charged multivesicular body protein 4b	CHMP4B	No
Q04837	Single-stranded DNA-binding protein, mitochondrial	SSBP1	No
P63244	Guanine nucleotide-binding protein subunit beta-2-like 1	GNB2L1	No*
A0A087X0Q1	YTH domain-containing family protein 3	YTHDF3	No
P07954	Fumarate hydratase, mitochondrial	FH	No*
P55072	Transitional endoplasmic reticulum ATPase	VCP	Yes*
H0YLP6	60S ribosomal protein L28	RPL28	No
P60880	Synaptosomal-associated protein 25	SNAP25	Yes*
P35232	Prohibitin	PHB	No*
O60506	Heterogeneous nuclear ribonucleoprotein Q	SYNCRIP	No
P27694	Replication protein A 70 kDa DNA-binding subunit	RPA1	No
F5GZS6	4F2 cell-surface antigen heavy chain	SLC3A2	No
P78371	T-complex protein 1 subunit beta	CCT2	Yes*
P17174	Aspartate aminotransferase, cytoplasmic	GOT1	No
O43765	Small glutamine-rich tetratricopeptide repeat-containing protein alpha	SGTA	No
P00441	Superoxide dismutase [Cu-Zn]	SOD1	No
A0A087X0X3	Heterogeneous nuclear ribonucleoprotein M	HNRNPM	No
P23246	Splicing factor, proline- and glutamine-rich	SFPQ	No*
A0A0A0-MR02	Voltage-dependent anion-selective channel protein 2	VDAC2	No
G3V203	60S ribosomal protein L18	RPL18	No

Continued on next page

Showing the identified substrates of LATS1 (cont.)			
**Protein IDs**	**Protein names**	**Gene names**	**Predicted**
A0A087X2D0	Serine/arginine-rich splicing factor 3	SRSF3	No
P16989	Y-box-binding protein 3	YBX3	No*
E9PK01	Elongation factor 1-delta	EEF1D	No
B5MCD7	Synaptogyrin-1	SYNGR1	No
P02763	Alpha-1-acid glycoprotein 1	ORM1	No
P67809	Nuclease-sensitive element-binding protein 1	YBX1	No*
A0A1W2-PPS1	Heterogeneous nuclear ribonucleoprotein U	HNRNPU	No
P07237	Protein disulfide-isomerase	P4HB	No
A0A087-WYT3	Prostaglandin E synthase 3	PTGES3	No
Q04917	14-3-3 protein eta	YWHAH	No
P41250	Glycine–tRNA ligase	GARS	No
P61978	Heterogeneous nuclear ribonucleoprotein K	HNRNPK	Yes*
Q8WUM4	Programmed cell death 6-interacting protein	PDCD6IP	No
E7EX17	Eukaryotic translation initiation factor 4B	EIF4B	No
P49368	T-complex protein 1 subunit gamma	CCT3	No
P16615	Sarcoplasmic/endoplasmic reticulum calcium ATPase 2	ATP2A2	No
O43175	D-3-phosphoglycerate dehydrogenase	PHGDH	No*
A6NMU3	Signal transducing adapter molecule 1	STAM	No
P60866	40S ribosomal protein S20	RPS20	No
P62854	40S ribosomal protein S26	RPS26	No
P27797	Calreticulin	CALR	No
E9PL71	Elongation factor 1-delta	EEF1D	No
Q15691	Microtubule-associated protein RP/EB family member 1	MAPRE1	No*
E9PEX6	Dihydrolipoyl dehydrogenase	DLD	No
Q92598	Heat shock protein 105 kDa	HSPH1	No
A0A0D9SFB1	Dynamin-1	DNM1	No
P11177	Pyruvate dehydrogenase E1 component subunit beta, mitochondrial	PDHB	No
Q99962	Endophilin-A1	SH3GL2	No*
P62753	40S ribosomal protein S6	RPS6	Yes*

Continued on next page

Showing the identified substrates of LATS1 (cont.)			
**Protein IDs**	**Protein names**	**Gene names**	**Predicted**
K7EMP8	Glial fibrillary acidic protein	GFAP	No
Q15366	Poly(rC)-binding protein 2	PCBP2	No
P26038	Moesin	MSN	No*
Q9Y5A9	YTH domain-containing family protein 2	YTHDF2	No
P51572	B-cell receptor-associated protein 31	BCAP31	No
D6RAF8	Heterogeneous nuclear ribonucleoprotein D0	HNRNPD	No
P04792	Heat shock protein beta-1	HSPB1	No*
E7EPN9	Protein PRRC2C	PRRC2C	No
P30040	Endoplasmic reticulum resident protein 29	ERP29	No
Q02790	Peptidyl-prolyl cis-trans isomerase FKBP4	FKBP4	No
F2Z388	60S ribosomal protein L35	RPL35	No
Q93077	Histone H2A type 1-C	HIST1H2AC	No
P23526	Adenosylhomocysteinase	AHCY	No
P27348	14-3-3 protein theta	YWHAQ	No*
P62879	Guanine nucleotide-binding protein G(I)/G(S)/G(T) subunit beta-2	GNB2	No
P00966	Argininosuccinate synthase	ASS1	No
Q5VTU8	ATP synthase subunit epsilon-like protein, mitochondrial	ATP5EP2	No
P05388	60S acidic ribosomal protein P0	RPLP0	No
Q9Y3F4	Serine-threonine kinase receptor-associated protein	STRAP	No*
O43852	Calumenin	CALU	No*
G3V4W0	Heterogeneous nuclear ribonucleoproteins C1/C2	HNRNPC	No
A0A0J9-YWU9	Ig heavy chain V-II region NEWM	IGHV4-61	No
B7ZKJ8	Inter-alpha-trypsin inhibitor heavy chain H4	ITIH4	No
P05023	Sodium/potassium-transporting ATPase subunit alpha-1	ATP1A1	No*
H7BZJ3	Protein disulfide-isomerase A3	PDIA3	No
P68871	Hemoglobin subunit beta	HBB	No
O15173	Membrane-associated progesterone receptor component 2	PGRMC2	No

Continued on next page

Showing the identified substrates of LATS1 (cont.)			
**Protein IDs**	**Protein names**	**Gene names**	**Predicted**
P38159	RNA-binding motif protein, X chromosome	RBMX	No*
P13804	Electron transfer flavoprotein subunit alpha, mitochondrial	ETFA	No
A0A087-WTT1	Polyadenylate-binding protein	PABPC1	No
P07737	Profilin-1	PFN1	No*
A0A0D9SFB3	ATP-dependent RNA helicase DDX3X	DDX3X	No
P17987	T-complex protein 1 subunit alpha	TCP1	No
U3KQ84	Dolichyl-diphosphooligosaccharide–protein glycosyltransferase 48 kDa subunit	DDOST	No
Q01650	Large neutral amino acids transporter small subunit 1	SLC7A5	No
P00558	Phosphoglycerate kinase 1	PGK1	No*
A6NLM8	Translocon-associated protein subunit delta	SSR4	No
P61081	NEDD8-conjugating enzyme Ubc12	UBE2M	No
P02749	Beta-2-glycoprotein 1	APOH	No
H3BV85	BolA-like protein 2	BOLA2B	No
A0A087-WUZ3	Spectrin beta chain, non-erythrocytic 1	SPTBN1	No
H3BRG4	Cytochrome b-c1 complex subunit 2, mitochondrial	UQCRC2	No
P51148	Ras-related protein Rab-5C	RAB5C	No
P08238	Heat shock protein HSP 90-beta	HSP90AB1	No*
Q9BZZ2	Sialoadhesin	SIGLEC1	No
Q9UQ80	Proliferation-associated protein 2G4	PA2G4	No*
P32119	Peroxiredoxin-2	PRDX2	No
P04844	Dolichyl-diphosphooligosaccharide–protein glycosyltransferase subunit 2	RPN2	No
P26373	60S ribosomal protein L13	RPL13	No*
P14625	Endoplasmin	HSP90B1	No*
Q9BVC6	Transmembrane protein 109	TMEM109	No
Q8WVC2	40S ribosomal protein S21	RPS21	No
P19338	Nucleolin	NCL	No*
P07900	Heat shock protein HSP 90-alpha	HSP90AA1	Yes*

Continued on next page

Showing the identified substrates of LATS1 (cont.)			
**Protein IDs**	**Protein names**	**Gene names**	**Predicted**
E7ETK0	40S ribosomal protein S24	RPS24	No
Q13185	Chromobox protein homolog 3	CBX3	No*
P26641	Elongation factor 1-gamma	EEF1G	No
F8VZJ2	Nascent polypeptide-associated complex subunit alpha	NACA	No
Q09028	Histone-binding protein RBBP4	RBBP4	No
P04083	Annexin A1	ANXA1	No*
P62888	60S ribosomal protein L30	RPL30	No
P10809	60 kDa heat shock protein, mitochondrial	HSPD1	No
P07195	L-lactate dehydrogenase B chain	LDHB	No
H0YHC3	Nucleosome assembly protein 1-like 1	NAP1L1	No
P50990	T-complex protein 1 subunit theta	CCT8	No
P00747	Plasminogen	PLG	No
H0Y7S3	Plasma membrane calcium-transporting ATPase 2	ATP2B2	No
P47756	F-actin-capping protein subunit beta	CAPZB	No
H0YMZ1	Proteasome subunit alpha type	PSMA4	No
P30050	60S ribosomal protein L12	RPL12	No*
O75367	Core histone macro-H2A.1	H2AFY	No
P09104	Gamma-enolase	ENO2	No
P62805	Histone H4	HIST1H4A	No
B4DJK0	Serine/arginine-rich splicing factor 5	SRSF5	No
P23396	40S ribosomal protein S3	RPS3	No*
P05387	60S acidic ribosomal protein P2	RPLP2	No
P36578	60S ribosomal protein L4	RPL4	No
J3QRS3	Myosin regulatory light chain 12A	MYL12A	No
P52597	Heterogeneous nuclear ribonucleoprotein F	HNRNPF	No
P0DP25	Calmodulin-3	CALM2	No
D6R9P3	Heterogeneous nuclear ribonucleoprotein A/B	HNRNPAB	No
P30153	Serine/threonine-protein phosphatase 2A 65 kDa regulatory subunit A alpha isoform	PPP2R1A	No
