# Supplementary figures and images for: Accurate prediction of kinase-substrate networks using knowledge graphs
Source: PLoS Comput Biol. 2020 Dec 3;16(12):e1007578. doi: 10.1371/journal.pcbi.1007578 (PMC7738173; doi:10.1371/journal.pcbi.1007578)

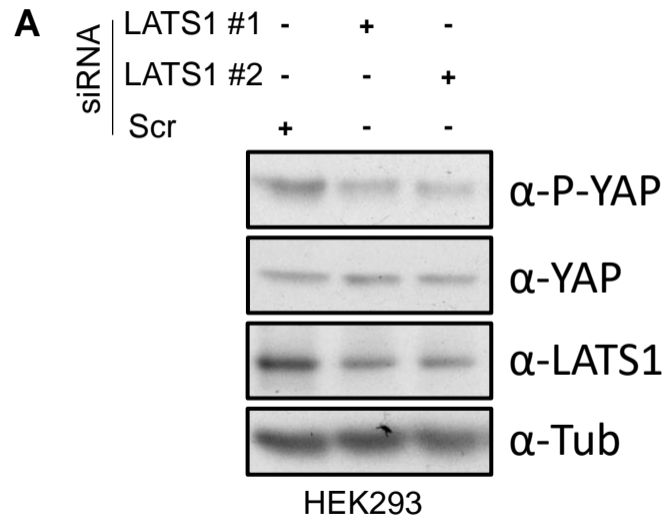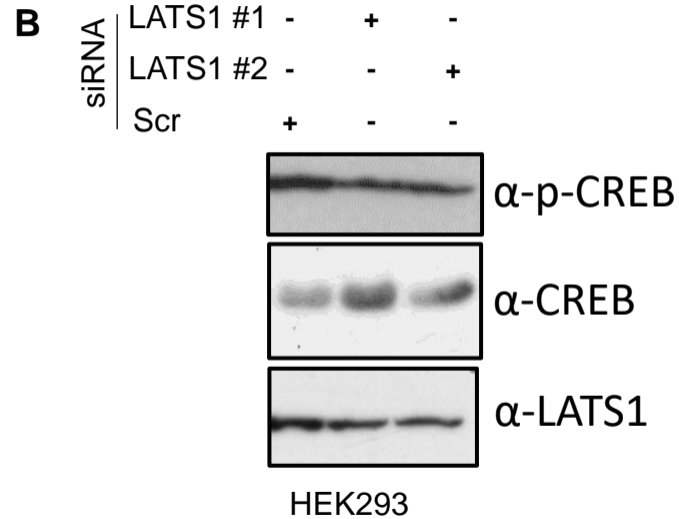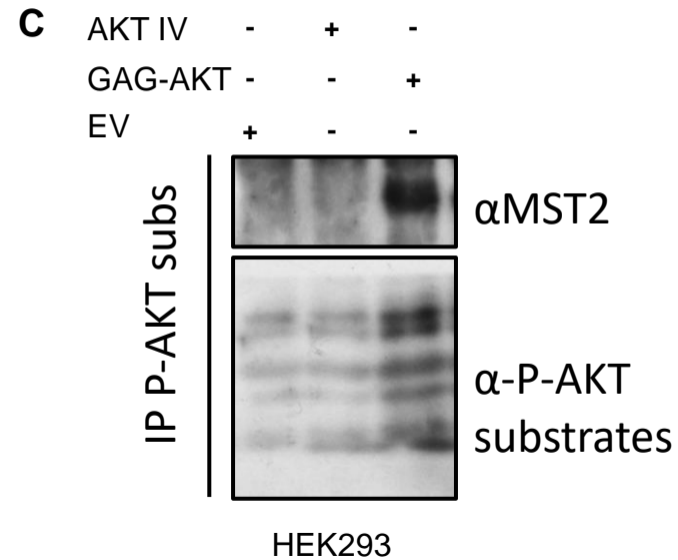

Supplement: S1 Fig — Supporting details on the experimental validation of the LATS1/YAP1 phosphorylation: (A-B) HEK293 were transfected with the indicated siRNAs. 48 hours after transfection the cells were lysed and blotted with the indicated antibodies. (C) HEK293 were transfected with empty vector (EV) or GAG-AKT or treated with AKTi IV (10M) for 1 hour. Phosphorylated proteins were immunoprecipitated using an anti-AKT antibody and the immunoprecipitates were blotted with the indicated antibodies (a PDF figure, c.f. https://doi.org/10.6084/m9.figshare.13118561). (PDF) [file pcbi.1007578.s010.pdf]
