# Supplementary material for: Accurate prediction of kinase-substrate networks using knowledge graphs
Source: PLoS Comput Biol. 2020 Dec 3;16(12):e1007578. doi: 10.1371/journal.pcbi.1007578 (PMC7738173; doi:10.1371/journal.pcbi.1007578)
Supplement: Attachment — Submitted filename: review_response_rev-2.pdf [file pcbi.1007578.s012.pdf]

Dear reviewer, editors,

we would like to thank you very much again for assessing our revised paper and providing us with further review comments - we have addressed them as described below.

The corresponding changes in the revised paper are highlighted in red font (note that there is quite a large “red area” in the figure legends, which is, however, only due to an addition of one extra figure as requested by the reviewer, and resulting changes in the numbering of the figures). We hope you will find this satisfactory and look forward to your feedback on this revision.

Detailed Responses to Reviewers’ Comments

We took the liberty of splitting the review comments into six specific remarks, to which we respond accordingly in the following paragraphs.

REMARK 1. *... I found it hard to navigate through the changes ...*

- We are sorry for the inconvenience of multiple spurious PDF copies, but this is how the submission system seems to have compiled the review PDF from the materials we submitted there based on the system guidelines.

REMARK 2. *Despite the fact that LinkPhinder outperforms all other methods, the poor results with the independent data-set, with AUPR and AUROC < 0.54 for all methods (Table 3), raises some substantial concerns regarding the performance of LinkPhinder in the internal validation (Table 1). It is important to understand if this is (i) an inherent problem of the validation data-set; (ii) potential overfitting of LinkPhinder to the test data-set; or (iii) methodological problem of the validation, e.g. data leakage from train to test.*

- We have tried to address this important issue in an extended comment after Table 3, but we elaborate on it here as well as this is probably the most important issue that needs to be properly clarified.
- One can easily verify in the data deposited at https://doi.org/10.1101/865055 that there is no leakage between positives (marked by 1 in the *y* column in the different splits) and negatives (marked by -1). Therefore point (iii) raised by the reviewer is not a likely explanation.
- Furthemore, we believe that the risk of potential overfitting (i.e. point (ii)) is rather low, as has been demonstrated by the additional experiments with different splits reported in the revised version (for details, see subsection 4.1.6 of the Materials and Methods part).
- This leaves us with point (i), which may indeed be true to some extent. The validation has inherent problems, the chief of which is the fact that it is impossible to train and test the various tools on exactly the same data, and also to make the related tools predict scores for negative statements. Thus one has to resort to proxies, such as the one we have proposed (this is all described in detail in Section 4.1.5).

- That doesn’t completely explain the different performance across different validation data, though. What we believe to be contributing to this is the following:
    - First of all, the performance of the tools appears to correlate with their coverage of the kinases represented in the benchmark – the higher the coverage, the better the results. Since the overlaps between the tools’ prediction sets and the Cutillas dataset are lower than for PhosphoSitePlus, it may partly explain the difference in the overall performance of the algorithms.
    - Moreover, the Cutillas data is extracted from only three cell lines, while the PhosphoSitePlus data covers many cell and tissue types, which may also partly explain the performance differences – if the tools are trained on existing knowledge that covers many different cell lines (like ours), it perhaps wouldn’t be a complete surprise for them to perform rather poorly on a dataset that is much more specific in terms of the cells covered.

REMARK 3. *Can the authors comment on the fact that PhosphoSitePlus is both used to train LinkPhinder and to generate the true positive sets for the AUPR and AUROC curves. This could lead to data leakage between train and test, i.e. training data is also used to test. Could the authors generate random sets of positive/negative interactions that are kept from LinkPhinder training and use them only for validation and comparison with other methods?*

- This has been done already in the first revision – see the previous comment for the exact reference to the corresponding data.

REMARK 4. *In addition, please report the total number of positive and negative statements (ideally have the list as supplementary material). ... Add details to the methods on how these are generated.*

- Similarly to the above, this has been done already. The lists for different splits and positive/negative ratios are a part of the supplementary materials, while the numbers of positives are reported in the text (for instance in Table 7, but also at various different places). The negatives are generated dynamically for each positive, (1 or 10 negatives per positive in the reported experiments, but any other number can be generated). Details on how the negatives are generated (among other things) are mentioned in Section 4.1.3.

REMARK 6. *Plot the AUPR and AUROC curves with the mean curve obtained for each model.*

- This is now addressed (in a new Figure 3).

This is it regarding our current revision. Thank you again, and should you have any further comments or requests, do not hesitate to contact me.

Best regards,

Vit (on behalf of all authors)
