# Supplementary material for: Accurate prediction of kinase-substrate networks using knowledge graphs
Source: PLoS Comput Biol. 2020 Dec 3;16(12):e1007578. doi: 10.1371/journal.pcbi.1007578 (PMC7738173; doi:10.1371/journal.pcbi.1007578)
Supplement: Attachment — Submitted filename: review_response_WK-updated.docx [file pcbi.1007578.s011.docx]

Dear reviewer, editors,

we would like to thank you very much for assessing our paper and providing us with review comments - these have been very useful for improving the presentation of our results and we have addressed them as described below.

The corresponding changes in the revised paper are highlighted in red font. In addition, to improve presentation and readability we also have moved the three large kinase assay tables towards the end of the manuscript into the supplementary information (not highlighted in red due to the amount of text moved and rather technical nature of this edit).

We hope you will find this satisfactory and look forward to your feedback on our revision.

**Detailed Responses to Reviewers’ Comments**

**RE. major review remarks**

*1. It would be important to exclude that the precisions reported are not inflated due to overfitting to the training data-set. Besides the 90-10% train-test split, can the authors use, for example, 60-40% and 80-20%?*

- This has been addressed in a corresponding comment in the Results part (p. 10), and a whole new subsection in the Materials and Methods part (4.1.6, p. 39-40).

*2. Taking advantage of a recent systematic study of kinase-substrate interactions (PMID:31959955), the authors could use this as an independent test set, while making sure the version of PhosphositePlus used for training does not contain this study to avoid circular training-testing. Can the authors show how well LinkPhinder captures these interactions and how it compares to the other approaches?*

- Very good point. We have used the recommended new dataset as a complementary benchmark and reported the results in a new table (3) and corresponding text (p. 13-14).

*3. LinkPhinder identifies 2,009,171 and 7,232,636 high and medium stringency interactions, respectively. It would be important to see overall statistics of the predicted kinase-substrate networks of each method, for example, the number of kinases covered, distribution and average number of predicted substrates per kinase, distribution and average number of kinases targeting each phosphorylation-site.*

- We have addressed this by a new figure (4) and text in the Results part (p. 12-13), and also added a complementary table (7) in the Materials and Methods part (p. 34), which reflects overlaps with respect to the dataset mentioned in the previous point.

*4. Some sections of the manuscript, for example section 2.3 and 2.4, have statements that are not supported with links to either figures, tables or supplementary material. I would recommend the authors to expand the number of figure panels accordingly to provide better support to the text. Also, data not shown statements are problematic to assess and reproduce. I would suggest either to provide the data as supplementary information or remove this statement.*

- In the initial presentation of our results we tried to be as complete as possible in reporting the results of our research, and therefore included description of data that were not shown. As recommended by the reviewers we now have either omitted such statements or supported them by showing the data. Specifically, we have made the following changes
- The experimental results of the AKT phosphorylation of SOS, which was described without showing the data, are now included in the supplementary information as Fig. S1
- The results reported under 2.3 are now included as a supplementary Fig. S1.
- In section 2.4 we used an unbiased biochemical method of global *in vitro* phosphorylation followed by MS based identification of phosphorylation sites to map the substrates of three specific kinases, PKA, LATS1 and MST2. The results for PKA and LATS1 are already included. The results for MST2 are now also included in the supplementary data as Fig. S1.

*5. Improved kinase-substrate networks are fundamental for methods that aim to estimate kinase activity profiles based on their substrates phosphorylation status. It would be interesting to see if LinkPhinder predicted network improves kinase activity predictions. An existing benchmark data-set (PMID:28200105) of quantitative phosphoproteomics measurements upon kinase inhibition could be used to test this.*

- We did address this point only partially by a new paragraph in the Discussion part (p. 25-26). The reason for such a decision is two-fold:
  a. Firstly, we have experimented with the code provided in the GitHub repo referenced in the paper (PMID:28200105) and found out that we could indeed extend the "regulons" data structure by our predicted substrates (after juggling the not entirely trivial issue of mapping the different ID schemes used, that is). However, populating the vector quantifying the corresponding sites seems to require a fair bit of novel research due to the many design decisions associated with using our data in a way compatible with the code. We therefore deemed this out of scope for this submission.
  b. Secondly, and more importantly, as stated in the newly included text, we do believe that such work, while certainly relevant and much needed, falls more under a follow-up of what we intended to achieve here. More concretely, this follow-up would be about showing how the better and more comprehensive predictions (as introduced in this submission) can help in different experiments aimed at the actual downstream functions of signalling cascades, which is no trivial matter that, in our opinion, deserves a dedicated publication.

**RE. minor review remarks**

*1. Is the predictive power of LinkPhinder networks significantly different between each run? How would LinkPhinder benchmark against other methods if instead of 100 runs of the experiment only one was performed?*

- This is now addressed by a new note in the Results part (p. 10).

*2. Table 1 could be complemented with plots of the AU-PR and AU-ROC as supplementary figures.*

- This is a valid remark, however, the plots are not bringing much added value over the data presented in the table. They are also potentially confusing due to the overlay of the 100 experimental runs, at least without a proper explanation that would be rather disruptive in the current flow of the section. Therefore we decided not to include them.

*3. Typo in “equal” in Table 1 legend.*

- This should be addressed now.

This is it regarding our revision. Thank you again, and should you have any further comments or requests, do not hesitate to contact me.

Best regards,

Vit (on behalf of all authors)
